# The effect of mobile-app-based instruction on the physical function of female patients with knee osteoarthritis: a parallel randomized controlled trial

**DOI:** 10.1186/s12905-021-01451-w

**Published:** 2021-09-14

**Authors:** Seyed Sajad Arfaei Chitkar, Hamid Reza Mohaddes Hakkak, Hassan Saadati, Seyed Hamid Hosseini, Yasaman Jafari, Reza Ganji

**Affiliations:** 1 School of Medicine, North Khorasan University of Medical Sciences, Bojnurd, Iran; 2 Department of Health Education and Health Promotion, School of Health, North Khorasan University of Medical Sciences, Bojnurd, Iran; 3 Department of Epidemiology and Biostatistics, School of Health, North Khorasan University of Medical Sciences, Bojnurd, Iran; 4 Department of Orthopedic Surgery, North Khorasan University of Medical Sciences, Bojnurd, Iran

**Keywords:** Osteoarthritis, Mobile app, Patients’ training

## Abstract

**Background:**

Osteoarthritis is a common disease and one of the most important causes of disability in the elderly that negatively affect the quality of their life. The purpose of this study was to evaluate the effectiveness of mobile app-based-instruction in improving physical performance of female patients with knee osteoarthritis.

**Methods:**

The present study was a randomized clinical trial. The sample included 64 female patients (40 to 70 years old) with knee osteoarthritis in Bojnurd city in 2018. They were selected from the available patients that were randomly divided into comparison and intervention groups. Before the intervention, demographic information questionnaire, Western Ontario and McMaster Universities Arthritis Index (WOMAC) questionnaire, and 36-item short-form health survey (SF-36) were employed to elicit data on demographic information, arthritis condition and health status of the participants. Intervention lasted for a period of two months for each group. Intervention group received mobile-app-based instruction coupled with routine cares, while comparison group just received the routine cares. After the intervention both groups were evaluated again in terms of arthritis condition and health status using the same scales.

**Results:**

After the intervention, significant differences were found between the intervention and comparison groups in terms of overall WOMAC score (*p* = 0.005), pain aspect of WOMAC (*p* = 0.005), physical function aspect of WOMAC (*p* = 0.005), physical function aspect of SF-36 (*p* ≤ 0.05), and vitality aspect of SF-36 (*p* > 0.05).

**Conclusion:**

The use of mobile-app-based instruction can enhance the physical function and quality of life in patients with knee osteoarthritis.

*Trial registration* The research project was registered at Iranian Registry of Clinical Trials (IRCT20161208031300N2).

## Background

Osteoarthritis (OA) is a common musculoskeletal disease that appears along with degenerative changes in the synovial joints and new bone formation [1]. It is the most common form of arthritis and joint disorder that adversely affects the public health [2, 3] and is the main known cause of disability. Recent estimations show that over 240 million people worldwide are suffering from this debilitating disease [4]. OA is commonly seen in pelvic, knees, spine, and finger joints[5]. However, knee OA is the most prevalent OA (33% prevalence) and has more clinical symptoms than any other forms of OA [6]. It is one of the five causes of disability in the elderly [7, 8]. In the United States, 14 million people are affected and more than half of them are under 65 [9]. American studies show that OA is on the rise [10]. Similarly, the prevalence of osteoarthritis seems to be increasing in Iran.

In epidemiological studies, including Community Oriented Program for Control of Rheumatic Diseases (COPCORD) the worldwide prevalence of knee osteoarthritis has been estimated 7%. The prevalence of OA in Iranian population over 15 years old has been estimated to be 16.9% and that of knee OA 15.5%. Regarding the prevalence of knee OA, Iran is ranked third among the studied countries[11, 12]. Osteoarthritis reduces mobility, quality of life, and well-being [13, 14], and increases health care and economic burden [15]. Unfortunately, the growing elderly population, sedentary lifestyle and obesity can add to the complications and the prevalence of OA. The prevalence of chronic osteoarthritis is predicted to increase and become the most common chronic skeletal disease in 2040 [16]. Aside from bone and joint damages, OA appears as a result of degeneration and destruction of the articular cartilage. It is usually seen in people over 40 and has slow progression rate as a result of repeated natural or abnormal pressures [17]. Knee osteoarthritis is caused by the incorrect performance of daily activities at younger age, as well as improper lifestyle and bodily movements [18]. Women account for a higher percentage of the OA patients [19] and female sex is a risk factor that increases the likelihood of developing OA [20]. Women experience greater incidence and more severity of OA than men, so it is necessary a greater need for effective treatment and prevention of OA in women[21, 22]. In addition, women often illustrate greater pain and more substantial reduction in function than men, which can lead to sarcopenia [23]. Lower extremity muscle appears to play a greater role in the development of knee OA in women than in men[24]. Although, the prevalence of OA is high, the population mean age is growing, and OA effects on mental and physical health [20], there are currently no methods to cure or stop the cartilage destruction [25]. Available treatment options are limited and at best can be effective on some patients [16]. The person with OA lives with it for 26 years on average [26, 27] and carries the burden of the disease for a long time. Not only the medical treatments, but also systematic health care is essential for proper treatment of OA [20]. Therefore, early diagnosis and continuous monitoring of OA progress are important in establishing effective treatment [28]. OA treatment goals include relieving pain and inflammation, lowering stiffness, improving function or range of motion, and improving or maintaining the mobility, physical activity, and health-related quality of life [29]. To achieve the treatment goals Patient education is needed. This is a continuous process and an integral part of patient management. The trainer should consider different aspects of the disease and the benefits and risks of treatment options. Empowering the patients by involving them in the decision-making process and teaching them skills for positive changes in life is a lengthy process for treatment [30]. This makes the individuals tired and may cause them to forget parts of their trainings. Therefore, patient education requires consistent monitoring of the patients’ performance. Due to decreased mobility and increased disability, the patient may not be able to visit the trainers in person and receive ongoing monitoring. Some believe that the available web-based approaches are more comfortable for the patients and can help them cope with the current shortage of skilled professionals and obstacles to ongoing face to face monitoring [12]. Dallimore et al. reports that the patients who used iPad scored significantly better in terms of recovery indices and were more satisfied with their treatment than other patients [31]. It has been also found that using technology tailored to users' needs lead to continued training in patients with OA and improve the motivational and behavioral factors [32]. A study has shown that the use of pedometer to provide feedback on walking activities of OA patients contributes to the attainment of OA treatment goals [33]. Another study found the use of smartphone app to be effective in training the OA patients and improving their condition [34].

Today, cell phones are part of everyday life, including health services. Mobile apps have the potentials to be exploited for medical purposes like instructing the patients for preventing diseases. In addition, the use of mobile apps can help for timely diagnosis and treatment, and lowering the costs of medical cares. Therefore, we decided to design a mobile app-based training program for patients with knee OA and evaluate its effectiveness on physical performance and behavioral change of these patients. Designing such apps allows for better monitoring and consistent instruction because it saves time and money, and frees the patients from the troubles of visiting their doctors in person. Proper and timely education can improve the quality of life in OA patients and reduce the incidence of knee osteoarthritis and its complications.

## Methods

The present study is a parallel randomized controlled trial. Participants of the study were 40–70 year-old female patients with knee osteoarthritis referring to Imam Ali Hospital in Bojnurd, affiliated to North Khorasan University of Medical Sciences. The sample size was determined through consulting previous similar studies [18, 35, 36]. Therefore, sample size was determined to consist of 28 people for each group using the G-power software with the confidence interval of 95% and the power of 80%. However, given the probability of a 10% drop in samples, 32 individuals were assigned to each group.

After determining the sample size, 64 female patients with knee osteoarthritis were selected from the referrals to Imam Ali Hospital in Bojnourd using the inclusion criteria. They were asked to sign the informed consent form before participating in the study. Patients were assigned to the intervention or comparison groups using blocked randomization method. In order to prevent information exchange between the intervention and comparison groups, patients were planned to be visited on different days. Prior to the intervention, patients were assessed in terms of demographic characteristics, OA condition and health status. To this end, demographic questionnaire, WOMAC, and SF-36 were completed for the patients in each group.

In order to observe blindness and avoid bias in data collection, the researcher who was collecting information on patients' performance and quality of life was kept unaware to which (intervention or comparison) groups the patients were belonging. Patients in the intervention group received all the educational content only through the mobile app. They were met only once at the beginning of the study to get familiar with using the app. Patients in the comparison group attended two face to face sessions; one introductory session at the beginning of the study and another one a month later of patients’ questions and further clarifications.

During the study, both groups received routine medical care. The educational content provided to both intervention and comparison groups included learning about OA and its causes, OA risk factors, the healthy diet and nutrients, treatment modalities, and the exercises for OA patients. Two months after the intervention, both groups were assessed again in terms of OA condition and health status using OWMAC and SF-36 respectively.

One patient from the intervention group and 3 patients from comparison group were excluded from the study because of discontinuing the treatment and participation. Finally, data from 60 patients (31 patients in the intervention group and 29 patients in the comparison group) were analyzed. Over an experimentation period was completed, the patients in the comparison group were also provided with the mobile app to get benefits from the instructions.

Inclusion criteria: (1) signing the informed consent form, (2) having the symptoms of knee osteoarthritis confirmed through radiologic assessment, (3) being 40 to 70 years old, (4) having a smartphone that is always available, (5) knowing how to use the mobile apps.

Exclusion criteria: (1) needing knee surgery during the study, (2) having intra-articular injections during the study, (3) suffering from mental illnesses, (4) unwilling to continue participation, (5) lacking the post-test data.

The data collection was done by employing three questionnaires: (a) demographic questionnaire that aimed to elicit data on the history of addiction, job, education, illness, age, BMI and marital status, (b) Western Ontario and McMaster Universities Arthritis Index (WOMAC) questionnaire consisting of 24 questions in three areas of pain, stiffness, and function of joints [35, 36], (c) Short form of health survey questionnaire (SF-36) by Weir and Sherborne (1992) consisting of 36 items and 8 components of Vitality, Physical Functioning, Bodily Pain, General Health, Physical Role Functioning, Emotional Role Functioning, Social Role Functioning, and Mental Health [37].

Data were analyzed by the student’s t test, and ANOVA using SPSS 18. The ANCOVA test was also used to adjust for the effects of the WOMAC and SF-36 scores at the baseline. Adjusted scores of WOMAC and SF-36 were reported as mean (SD). Significance level to interpret the statistical results was set at *p* ≤ 0.05.

## Results

**Fig. 1:**
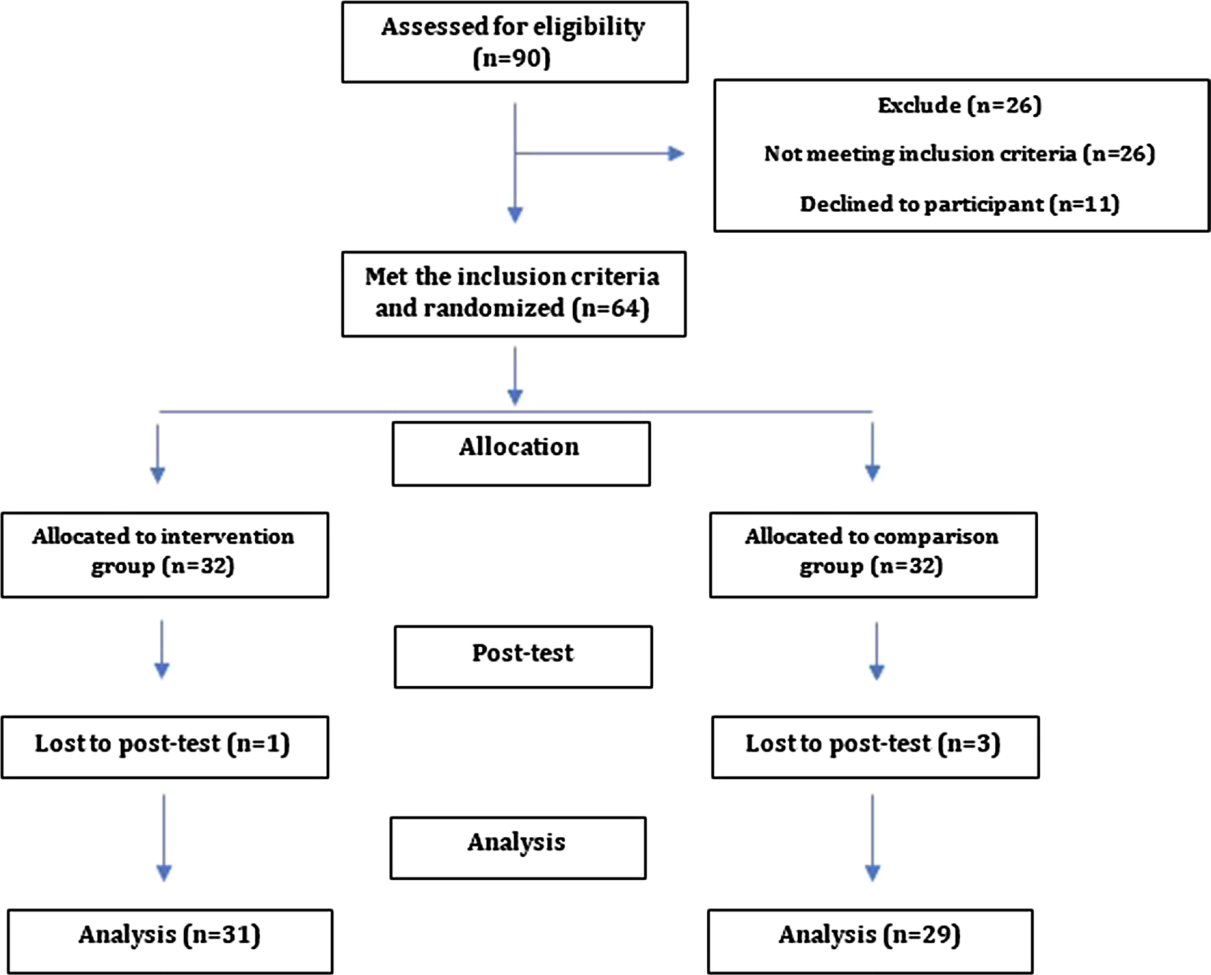
Flow diagram of the present study

The flow diagram of participants in this clinical trial is shown in Fig. 1. Initially, 90 patients were included in the study using the inclusion criteria. However, 15 patients who were later found not eligible and 11 patients who were not willing to participate were excluded. Finally, the sample consisted of 64 female patients with knee osteoarthritis who were randomly assigned to two equal groups (intervention and comparison groups). The baseline characteristics of the participants are shown in Table 1. The results of the chi-square test showed that there was no significant difference between the two groups in terms of age, BMI, history of addiction, occupation, co-morbidity, duration of illness and education (*p* < 0.05). Table 2 shows the means and standard deviations of the WOMAC and SF-36 questionnaires at the beginning of the study. The mean scores of intervention and comparison groups in general health were 18.67 (SD = 10.97) and 25.48 (SD = 13.17), respectively, and were significantly different before the intervention (*p* = 0.035). There was no statistically significant difference between the two groups in other areas of WOMAC and SF-36 (*p* > 0.05). Table 3 shows the, the means of crude d and adjusted scores (both total and sub scores) in physical performance (WOMAC) and quality of life (SF-36) after the intervention. The adjusted *p* values are also shown in Table 3. It was observed that after the intervention there were significant differences between the intervention and comparison groups in total, pain, and functional limitation scores of WOMAC (*p* < 0.005) except the stiffness (*p* = 0.618). Also, the results did not change after adjusting for baseline values using ANCOVA test. The comparisons of the groups after the intervention in terms of the components of quality of life (SF-36) showed that the two groups were significantly different only in physical functioning and energy/fatigue. Physical functioning and energy/fatigue scores in the intervention group were 41.45 (8.68) and 67.09 (11.74) respectively, and in the comparison, group were 32.41 (7.51) and 56.72 (10.46) respectively (*p* < 0.005). After adjusting for baseline values, the difference between the two groups was also significant in the pain component (*p* = 0.047). Overall, it was concluded that the mobile app-based training program improves the physical performance of the intervention group compared to the comparison group.

**Table 1:** Participant characteristics at baseline

Variable	Total (n = 60)	Intervention group (n = 31)	Comparison group (n = 29)
Age, mean (SD)	58.17 (7.55)	57.84 (8.63)	58.52 (6.33)
BMI, mean (SD)	27.31 (3.98)	27.97 (4.44)	26.62 (3.37)
History of addiction, No. (%)	32 (53.3)	19 (61.3)	13 (44.8)
Job, No. (%)			
Housekeeper	51 (85)	26 (83.9)	25 (86.2)
Employee	7 (11.7)	4 (12.9)	3 (10.3)
Free job	2 (3.3)	1 (3.2)	1 (3.4)
Type of comorbidity, No. (%)			
No	12 (20)	7 (22.6)	5 (17.2)
HLP	9 (15)	1 (3.2)	8 (27.6)
HTN	21 (35)	12 (38.7)	9 (31)
ASTHMA	4 (6.7)	3 (9.7)	1 (3.4)
C.P	1 (1.7)	1 (3.2)	–
Lumber pain	13 (21.7)	7 (22.6)	6 (20.7)
Duration of catch			
1–3 years	2 (3.33)	2 (6.5)	–
3–6 years	7 (11.7)	4 (12.9)	3 (10.3)
6–10 years	49 (81.6)	25 (80.6)	24 (82.8)
10–15 years	2 (3.33)	–	2 (6.9)
Education, No. (%)			
Illiterate	45 (75)	22 (71)	23 (79.3)
Diploma	11 (18.3)	6 (19.4)	5 (17.2)
Associate Degree	4 (6.7)	3 (9.7)	1 (3.4)
Married, No. (%)	60 (100)	31 (100)	29 (100)

**Table 2:** Mean scores for SF-36 and WOMACK at the baseline

Domain	Average scores*, mean (SD)		
	Intervention group (n = 31)	Comparison group (n = 29)	*P* value
WOMAC			
Pain	18.45 (5.10)	17.17 (1.69)	0.204
Stiffness	5.87 (1.05)	5.58 (0.77)	0.242
Functional limitation	48.32 (4.21)	49.61 (5.24)	0.333
Total	72.64 (7.15)	72.27 (5.53)	0.825
SF-36			
Physical functioning	27.25 (8.83)	27.41 (7.97)	0.943
Role limitations due to physical health	12.09 (15.64)	13.79 (14.30)	0.663
Role limitations due to emotional problems	27.95 (24.49)	18.39 (22.86)	0.124
Energy/fatigue	50.96 (13.50)	49.82 (11.21)	0.724
Emotional well being	60 (8.94)	56.82 (10.33)	0.280
Social functioning	36.69 (15.11)	38.36 (15.24)	0.672
Pain	40.40 (17.98)	36.55 (17.28)	0.402
General health	18.67 (10.97)	25.48 (13.17)	0.035

*Baseline measurement

**Table 3:** Comparison of the mean scores for SF-36 and WOMACK after intervention

Domain	Crude scores, mean (SD)			Adjusted scores, mean (SE		
	Intervention group (n = 31)	Control group (n = 29)	*p* value	Intervention group (n = 31)	Control group (n = 29)	*p* value
WOMAC						
Pain	11.80 (1.32)	13.82 (1.46)	0.0001	11.79 (0.25)	13.84 (0.26)	0.0001
Stiffness	4.80 (0.98)	4.68 (0.80)	0.618	4.73 (0.14)	4.76 (0.14)	0.895
Functional limitation	41 (3.54)	45.86 (4.83)	0.0001	41.45 (0.37)	45.38 (0.38)	0.0001
Total	57.61 (3.87)	64.37 (4.85)	0.0001	57.53 (0.61)	64.46 (0.63)	0.0001
SF-36						
Physical functioning	41.45 (8.68)	32.41 (7.51)	0.0001	41.49 (1.19)	32.37 (1.23)	0.0001
Role limitations due to physical health	12.09 (15.60)	13.79 (14.30)	0.663	12.91 (0.0001)	12.91 (0.0001)	0.054
Role limitations due to emotional Problems	27.95 (24.49)	18.39 (22.86)	0.124	29.74 (4.46)	16.48 (4.62)	0.054
Energy/fatigue	67.09 (11.74)	56.72 (10.46)	0.001	66.72 (1.31)	57.12 (1.35)	0.0001
Emotional well being	60 (8.94)	56.82 (10.33)	0.208	58.46 (0.0001)	58.46 (0.0001)	0.99
Social functioning	40.32 (15.38)	40.51 (14.03)	0.959	40.89 (1.81)	39.90 (1.88)	0.706
Pain	51.61 (15.75)	44.13 (17.14)	0.084	50.10 (1.48)	45.74 (1.53)	0.047
General health	26.25 (11.70)	31 (11.32)	0.117	29.3 (1)	28.04 (1.03)	0.504

## Discussion

The purpose of this study was to evaluate the efficacy of mobile app-based-instruction in improving physical function of female patients with knee osteoarthritis. The results showed that using mobile app-based-instruction significantly reduces pain, and improves the physical function and overall condition of patients with knee osteoarthritis. It also improved some aspects of quality of life, such as fatigue/energy, physical function and pain in intervention group compared to the comparison group. These results are in line with those of Danbjørg et al. [32], Dallimore et al. [31], Skrepnik et al. [34], Semple et al. [38], Brun Thorup et al. [33]. In these studies, an application or a web-based service was used to educate and monitor the recovery process of the patients. We found no studies pointing to the ineffectiveness of mobile apps in improving the physical performance of patients with OA.

Certain explanations can be put forward on the effect of mobile app-based instruction in physical performance, pain perception and some aspects of health in patients with knee OA. First, mobile app-based instruction provides easy access to reliable information. Efficient health care requires accurate and useful information which is difficult to obtain at times. Despite the information available in the twenty-first century, people may not be able to get access to reliable and credible information. This justifies the attempts for direct access to a specialist for proper instructions. On the other hand, visiting a doctor frequently has been time-consuming and difficult for many OA patients. The educational app, not only makes it easy to access educational services, but also provides reliable and credible information. As a result, it increases the likelihood of the use of educational content by the patients. Second, mobile apps can be used at any time and place. Smartphones enable the patients to read the instructions and use them whenever they want. Third, mobile apps make the patients more motivated to exercise and monitor their performance because mobile apps can have built-in mechanisms for allowing the patients to monitor their status and responding to their questions. Fourth, people are increasingly getting accustomed to use mobile apps. This growing habit of frequent use of mobile apps raises the effectiveness of mobile apps in instructing the patients.

## Conclusion

Based on the results of the present study and related studies, it can be concluded that using mobile app-based instructions can significantly reduce the pain and improve some health aspects in women with knee OA. Accordingly, it is useful in medical, clinical and physiological management of knee osteoarthritis. Such benefits of mobile app-based instructions can have positive effects on the physical and mental health of people with knee OA and increase their satisfaction and quality of life. As a result, mobile app-based instruction can be used to educate patients while saving their time and money. It is suggested that the benefits of mobile app-based instruction in other diseases be further studied. Due to fact statistical significance was not found in relation to the effect of mobile app-based instruction on some aspects of quality of life (as measured by SF-39), it is recommended that efficacy of mobile app-based instruction in improving different aspects of quality of life be further studied for longer periods of life.

## Data Availability

The datasets used and/or analyzed during the current study are available from the corresponding author on reasonable request.

## Abbreviations

WOMAC: Western Ontario and McMaster Universities Arthritis Index
OA: Osteoarthritis
COPCORD: Community Oriented Program for Control of Rheumatic Diseases

## References

[1] Silva ALP, Imoto DM, Croci AT (2007). Comparison of cryotherapy, exercise and short waves in knee osteoarthritis treatment. Acta Ortopédica Brasileira.

[2] Nelson AE (2018). Osteoarthritis year in review 2017: clinical. Osteoarthritis Cartilage.

[3] Cross M, Smith E, Hoy D, Nolte S, Ackerman I, Fransen M, Bridgett L, Williams S, Guillemin F, Hill CL (2014). The global burden of hip and knee osteoarthritis: estimates from the global burden of disease 2010 study. Ann Rheum Dis.

[4] Vos T, Allen C, Arora M, Barber RM, Bhutta ZA, Brown A, Carter A, Casey DC, Charlson FJ, Chen AZ (2016). Global, regional, and national incidence, prevalence, and years lived with disability for 310 diseases and injuries, 1990–2015: a systematic analysis for the Global Burden of Disease Study 2015. The Lancet.

[5] Mathers DSC, Pfleger B: Global burden of osteoarthritis in the year 2000. World Health Organization, www.whoint/healthinfo/statistics/bod_osteoarthritispdf2003.

[6] Deyle GD, Allison SC, Matekel RL, Ryder MG, Stang JM, Gohdes DD, Hutton JP, Henderson NE, Garber MB (2005). Physical therapy treatment effectiveness for osteoarthritis of the knee: a randomized comparison of supervised clinical exercise and manual therapy procedures versus a home exercise program. Phys Ther.

[7] Jordan JM, Linder GF, Renner JB, Fryer JG (1995). The impact of arthritis in rural populations. Arthritis Rheum.

[8] DeChellis DM, Cortazzo MH (2011). Regenerative medicine in the field of pain medicine: prolotherapy, platelet-rich plasma therapy, and stem cell therapy—theory and evidence. Tech Region Anesth Pain Manag.

[9] Deshpande BR, Katz JN, Solomon DH, Yelin EH, Hunter DJ, Messier SP, Suter LG, Losina E (2016). Number of persons with symptomatic knee osteoarthritis in the US: impact of race and ethnicity, age, sex, and obesity. Arthritis Care Res.

[10] Barbour KE, Helmick CG, Boring M, Brady TJ (2017). Vital signs: prevalence of doctor-diagnosed arthritis and arthritis-attributable activity limitation—United States, 2013–2015. MMWR Morb Mortal Wkly Rep.

[11] Davatchi F, Sandoughi M, Moghimi N, Jamshidi AR, Tehrani Banihashemi A, Zakeri Z, Sadeghi Abdollahi B (2016). Epidemiology of rheumatic diseases in Iran from analysis of four COPCORD studies. Int J Rheum Dis.

[12] Lespasio MJ, Piuzzi NS, Husni ME, Muschler GF, Guarino A, Mont MA: Knee osteoarthritis: a primer. Permanente J 2017, 21. 10.7812/TPP/16-183 PMC5638628 29035179

[13] Schlenk EA, Lias JL, Sereika SM, Dunbar-Jacob J, Kwoh CK (2011). Improving physical activity and function in overweight and obese older adults with osteoarthritis of the knee: a feasibility study. Rehabil Nurs.

[14] Etesami AS, Zamani J, Zolaktaf V, Ghasemi G (2015). Effectiveness of aquatic exercise therapy on the quality of life in women with knee osteoarthritis. Iran J Ageing.

[15] Ackerman IN, Ademi Z, Osborne RH, Liew D (2013). Comparison of health-related quality of life, work status, and health care utilization and costs according to hip and knee joint disease severity: a national Australian study. Phys Ther.

[16] Van Spil WE, Kubassova O, Boesen M, Bay-Jensen A-C, Mobasheri A: Osteoarthritis phenotypes and novel therapeutic targets. Biochem Pharmacol 2019. 10.1016/j.bcp.2019.02.037 30831073

[17] Sowers M (2001). Epidemiology of risk factors for osteoarthritis: systemic factors. Curr Opin Rheumatol.

[18] Mirzaee N, Mohammadi-Shahbolaghi F, Nowroozi K, Biglarian A, Rangin H (2016). The effect of self-management training on performance of elderly patients with knee osteoarthritis. Iran J Nurs.

[19] Park JI, Jung HH (2017). Estimation of years lived with disability due to noncommunicable diseases and injuries using a population-representative survey. PLoS ONE.

[20] Vina ER, Kwoh CK (2018). Epidemiology of osteoarthritis: literature update. Curr Opin Rheumatol.

[21] O'Connor MI (2006). Osteoarthritis of the hip and knee: sex and gender differences. Orthop Clin.

[22] Srikanth VK, Fryer JL, Zhai G, Winzenberg TM, Hosmer D, Jones G (2005). A meta-analysis of sex differences prevalence, incidence and severity of osteoarthritis. Osteoarthritis Cartilage.

[23] Glass N, Segal N, Sluka K, Torner J, Nevitt M, Felson D, Bradley L, Neogi T, Lewis C, Frey-Law L (2014). Examining sex differences in knee pain: the multicenter osteoarthritis study. Osteoarthritis Cartilage.

[24] Segal NA, Torner JC, Felson D, Niu J, Sharma L, Lewis CE, Nevitt M (2009). Effect of thigh strength on incident radiographic and symptomatic knee osteoarthritis in a longitudinal cohort. Arthritis Care Res.

[25] March L, Cross M, Lo C, Arden N, Gates L, Leyland K, Hawker G, King L: Osteoarthritis: a serious disease. OARSI org https://www.oarsi.org/sites/default/files/docs/2016/oarsi_white_paper_oa_serious_disease_121416_1pdf2016.

[26] Losina E, Weinstein AM, Reichmann WM, Burbine SA, Solomon DH, Daigle ME, Rome BN, Chen SP, Hunter DJ, Suter LG (2013). Lifetime risk and age at diagnosis of symptomatic knee osteoarthritis in the US. Arthritis Care Res.

[27] Simon LS (2012). Relieving pain in America: A blueprint for transforming prevention, care, education, and research. J Pain Palliat Care Pharmacother.

[28] Stolz M, Gottardi R, Raiteri R, Miot S, Martin I, Imer R, Staufer U, Raducanu A, Düggelin M, Baschong W (2010). Early detection of aging cartilage and osteoarthritis in mice and patient samples using atomic force microscopy. Nat Nanotechnol.

[29] Hafez AR, Alenazi AM, Kachanathu SJ, Alroumi A, Mohamed E (2014). Knee osteoarthritis: a review of literature. Phys Med Rehabil Int.

[30] Swięchowicz S, Ostałowska A, Kasperczyk A, Nowak D, Birkner E, Kasperczyk S (2012). Evaluation of hyaluronic acid intra-articular injections in the treatment of primary and secondary osteoarthritis of the knee. Pol Orthop Traumatol.

[31] Dallimore R-K, Asinas-Tan ML, Chan D, Hussain S, Willett C, Zainuldin R (2017). A randomised, double-blinded clinical study on the efficacy of multimedia presentation using an iPad for patient education of postoperative hip surgery patients in a public hospital in Singapore. Singapore Med J.

[32] Danbjørg DB, Villadsen A, Gill E, Rothmann MJ, Clemensen J (2018). Usage of an exercise app in the care for people with osteoarthritis: user-driven exploratory study. JMIR Mhealth Uhealth.

[33] Brun Thorup C, Grønkjær M, Spindler H, Andreasen JJ, Hansen J, Dinesen BI, Nielsen G, Sørensen EE: Pedometer use as motivation for physical activity in cardiac tele-rehabilitation. Int J Integr Care 2015, 15(7).

[34] Skrepnik N, Spitzer A, Altman R, Hoekstra J, Stewart J, Toselli R (2017). Assessing the impact of a novel smartphone application compared with standard follow-up on mobility of patients with knee osteoarthritis following treatment with Hylan GF 20: a randomized controlled trial. JMIR Mhealth Uhealth.

[35] Bellamy N, Buchanan WW, Goldsmith CH, Campbell J, Stitt LW (1988). Validation study of WOMAC: a health status instrument for measuring clinically important patient relevant outcomes to antirheumatic drug therapy in patients with osteoarthritis of the hip or knee. J Rheumatol.

[36] Ebrahimzadeh MH, Makhmalbaf H, Birjandinejad A, Keshtan FG, Hoseini HA, Mazloumi SM (2014). The Western Ontario and McMaster Universities Osteoarthritis Index (WOMAC) in persian speaking patients with knee osteoarthritis. Arch Bone J Surg.

[37] Montazeri A, Goshtasbi A, Vahdaninia M. The short form health survey (SF-36): translation and validation study of the Iranian version (2006). 10.1007/s11136-004-1014-5 16022079

[38] Semple JL, Sharpe S, Murnaghan ML, Theodoropoulos J, Metcalfe KA (2015). Using a mobile app for monitoring post-operative quality of recovery of patients at home: a feasibility study. JMIR Mhealth Uhealth.

