# Correction to: The effect of mobile-app-based instruction on the physical function of female patients with knee osteoarthritis: a parallel randomized controlled trial

**DOI:** 10.1186/s12905-021-01491-2

**Published:** 2021-10-05

**Authors:** Seyed Sajad Arfaei Chitkar, Hamid Reza Mohaddes Hakkak, Hassan Saadati, Seyed Hamid Hosseini, Yasaman Jafari, Reza Ganji

**Affiliations:** 1 School of Medicine, North Khorasan University of Medical Sciences, Bojnurd, Iran; 2 Department of Health Education and Health Promotion, School of Health, North Khorasan University of Medical Sciences, Bojnurd, Iran; 3 Department of Epidemiology and Biostatistics, School of Health, North Khorasan University of Medical Sciences, Bojnurd, Iran; 4 Department of Orthopedic Surgery, North Khorasan University of Medical Sciences, Bojnurd, Iran

## Correction to: BMC Women’s Health (2021) 21:333 10.1186/s12905-021-01451-w

Following the publication of the original article [1], we were notified that Table 2 was published as a duplicate of Table 1. Table 2 has now been replaced with the respective data set.

The original article has been corrected.
